# Breathing New Life Into Donation After Circulatory Death (DCD) Lung Transplantation: The Role of Machine Perfusion

**DOI:** 10.7759/cureus.87196

**Published:** 2025-07-02

**Authors:** Nisarg N Gandhi, Julie DeSantis, Jeffrey A Miskoff

**Affiliations:** 1 Department of Urology, Division of Transplant, The University of Toledo College of Medicine and Life Sciences, Toledo, USA; 2 Clinical Transplant, NJ Sharing Network, New Providence, USA

**Keywords:** donation after circulatory death (dcd), ex-vivo lung perfusion, lung transplantation, normothermic machine perfusion, organ preservation, organ procurement

## Abstract

Lung transplantation (LTx) is a life-saving procedure for patients with end-stage pulmonary disease, but the shortage of suitable donor lungs remains a critical barrier. Traditionally, lungs from donors after brain death (DBD) have been preferred, though the growing presence of donation after circulatory death (DCD) is a viable solution to expand the donor pool. However, DCD lungs present unique challenges, primarily due to exposure to warm ischemia, higher aspiration risk, hurried evaluation, and the lack of real-time viability assessment. Additionally, many donor hospitals and organ procurement organizations (OPOs) impose limitations on certain pre-mortem interventions, such as restrictions on aggressive lung recruitment, a potential barrier to improvement of lung function, and bronchoscopy, which can reduce the willingness of transplant centers to accept lung offers. This review examines the impact of modern preservation methods, particularly machine perfusion (MP) technologies such as ex vivo lung perfusion (EVLP) and the Organ Care System (OCS), on DCD lung transplantation outcomes. MP technologies allow for the preservation, assessment, and reconditioning of DCD lungs, significantly improving their viability and transplantation success rates. Studies indicate that MP can reduce the risks of ischemia-reperfusion injury (IRI), enhance graft function, and increase lung utilization without compromising short-term or long-term survival outcomes. Despite this, the implementation of MP faces challenges related to logistical coordination, cost-effectiveness, and protocol variability. With that said, the growing body of evidence suggests that MP can help to overcome the traditional limitations of DCD lungs, thus expanding the donor pool and improving transplant outcomes.

## Introduction and background

Lung transplantation (LTx) remains a critical, life-saving intervention for patients with end-stage pulmonary disease. However, a persistent shortage of suitable donor lungs limits its potential impact, with many patients dying while on the waiting list. Traditionally, lungs procured from donors after brain death (DBD) have been preferred for transplantation, though in recent years, donation after circulatory death (DCD) has emerged as a promising alternative in expanding the donor pool as it becomes more routine [1]. Although using lungs from DCD donors can be challenging due to warm ischemia and limited assessment of lung function in real time, such as serial arterial blood gas (ABG) analyses, unless thoracoabdominal normothermic regional perfusion (TA-NRP) is being used, machine perfusion (MP) technologies have made it possible to safely use these donor lungs. Controlled DCD (cDCD), which is the process of organ donation occurring after a planned withdrawal of mechanical ventilation, has demonstrated significant potential to minimize the discrepancy between organ supply and demand [2].

Moreover, MP technologies, such as ex vivo lung perfusion (EVLP) and the Organ Care System (OCS) Lung device, have been shown to mitigate limitations of DCD lungs by maintaining them in a normothermic, near-physiologic condition and allowing for real-time evaluation and reconditioning of marginal lungs [3,4]. These modern preservation modalities ultimately reduce discard rates and improve the chances of successful transplants [5]. Notably, the single-arm pivotal EXPAND trial demonstrated that 81 (87%) of 93 extended-criteria lungs preserved using the portable OCS Lung system were successfully transplanted, with excellent short-term outcomes [4,6]. EVLP has further been recognized for its role in salvaging lungs initially deemed unsuitable, increasing the donor lung pool without compromising graft function [7].

In addition, comparative studies suggest that the use of MP does not compromise long-term survival. A five-year follow-up from the International Society for Heart and Lung Transplantation (ISHLT) Registry reported comparable survival outcomes between DCD and DBD recipients [8]. Warm ischemic times (WITs) up to 60 minutes did not negatively impact one-year survival [9]. Given the accumulating evidence and technological advancements, we conducted a narrative review to investigate the way in which MP technologies impact transplantation rates, organ utilization, and post-transplant outcomes in DCD LTx as compared to conventional static cold storage (SCS). Of note, since this is a narrative review, no pooling took place.

## Review

Barriers to the implementation of DCD LTx

Despite the growing interest in DCD organ donation as a strategy to expand the donor pool, several clinical and logistical barriers continue to limit its widespread adoption in lung transplantation.

Risk of Primary Graft Dysfunction

One major clinical concern is the risk of primary graft dysfunction (PGD), an acute lung injury syndrome occurring within 72 hours post-transplant, often triggered by ischemia-reperfusion injury (IRI). DCD lungs have historically shown higher PGD rates, largely due to warm ischemia. However, the implementation of EVLP and portable organ care systems has significantly reduced this risk. According to Wagner et al., long-term outcomes of lungs treated with EVLP have been comparable to DBD lungs in selected settings, despite those lungs being characterized by more risk factors for transplant failure [10].

Warm Ischemic Time and Ischemia-Reperfusion Injury

Unlike DBD donation, DCD procurement involves a variable agonal phase after withdrawal of life support, during which warm ischemia may prolong. Extended WIT correlates with poorer early graft function and heightened risk of PGD. The resulting IRI contributes to pulmonary endothelial damage, further exacerbating PGD risk. Meta-analyses and registry data, including from the ISHLT Registry, link prolonged WIT and insufficient protective strategies to higher PGD rates and early graft dysfunction in DCD recipients [11].

Protocol Variability and Lack of Standardization

Inter-center variability in DCD protocols, including donor selection criteria and acceptable agonal durations, complicates outcome comparisons and impedes consensus. Ahmad et al. and Van Raemdonck et al. highlighted that the absence of standardized thresholds for WIT and procurement practices limits reproducibility and generalizability [8,12].

Additional Barriers: Ethical, Logistical, and Economic

Beyond clinical concerns, ethical debates over pre-mortem interventions (such as recruitment or bronchoscopy), logistical complexity in coordinating cDCD timing, and cost implications of flying out, traveling for a DCD that is ultimately aborted present further obstacles.

Machine perfusion technologies

Innovative MP technologies have challenged the aforementioned barriers. By addressing core limitations, such as ischemic injury and organ viability, these advancements are not only enhancing graft outcomes but also redefining what is possible in DCD LTx, increasing utilization while minimizing post-procurement discard rates. Additionally, MP technologies facilitate logistical flexibility.

Ex vivo Lung Perfusion Technologies

EVLP represents a major advancement in donor lung preservation and assessment. This technology allows lungs to be perfused and ventilated in a normothermic fashion, enabling continuous monitoring of function, gas exchange, compliance, and vascular resistance. EVLP provides a unique opportunity to rehabilitate marginal lungs. EVLP, when used as a "bridge" after static cold storage, allows clinicians to reassess donor lungs previously deemed unsuitable [12,13]. Also, by simulating physiologic conditions, EVLP lowers the risk and severity of reperfusion injury: creating a controlled, normothermic environment enables gradual re-oxygenation and perfusion, reducing the endothelial damage associated with IRI, in turn reducing risk to recipients [14,15]. Similarly, Wagner et al. found that EVLP significantly reduces the risk of PGD, leading to comparable or improved post-transplant outcomes relative to conventional methods [10].

XVIVO Perfusion System

The XVIVO Perfusion System (XPS; XVIVO, Gothenburg, Sweden), an EVLP system using the STEEN Solution as its perfusion solution, is widely employed in centralized lung re-conditioning hubs, particularly in Europe and North America. Unlike the portable OCS, XPS is typically used in a cold-to-warm approach following conventional static storage, as mentioned above. On arrival at the transplant center, lung(s) are placed on the XPS system with the use of perfusion cannulas and an endotracheal tube, and evaluated for up to six hours.

Though a static platform compared to OCS, its utility in lung evaluation and rehabilitation makes it a valuable assessment platform. As referenced by Ahmad et al. and Loor and Sanchez, by allowing for the evaluation of lung function up to four to six hours after procurement, the XPS system increased confidence in marginal lung selection, increasing lung transplant volume by up to 20% by accepting organs that were initially declined [12,14].

TransMedics Organ Care System Lung

Another popular EVLP technology, OCS Lung by TransMedics Inc. (MA, USA) offers portable, normothermic perfusion during transportation, maintaining the lung in a near-physiological state. The EXPAND trial and subsequent long-term data demonstrated that the OCS significantly increases the utilization rate of DCD and ECD lungs without compromising recipient outcomes [4]. Loor and colleagues found that 79 of 91 lung pairs (87%) of ECD lungs that were placed on OCS Lung were successfully transplanted, with a one-year survival rate of 91% for patients who received these lung transplants [4]. OCS also allows for long-distance retrieval, offering logistical flexibility for transplant centers with a limited local lung donor population.

Paragonix BAROguard

While not technically MP, the advanced preservation capabilities of the Paragonix BAROguard System (Paragonix Technologies, Inc., MA, USA) introduced improvements over conventional ice storage by providing pressure- and variable temperature-controlled (4-8°C) hypothermic preservation. This system mitigates risks associated with cold-induced injury by maintaining a controlled variable thermal environment of 4-8°C, preventing overcooling.

Initial studies report that BAROguard shows promising results in maintaining lung viability and reducing PGD incidence, and is particularly useful for centers that have limited means of machine perfusion, improving outcomes of DCD lung procurement [16,17]. Its implementation represents a meaningful step forward in preservation of donor lungs, without the infrastructure and capital required for EVLP or OCS. With that said, the risk of IRI remains, as it is hypothermic storage.

Conventional static cold storage

SCS remains the most commonly used method of lung preservation due to its simplicity, portability, and low cost. Lungs are typically flushed with a preservation solution and stored in a three-layered bag system, each separated by fluid, and then placed in an ice cooler for hypothermic transport. This reduces metabolic activity and limits oxygen demand, in turn potentially limiting associated ischemic insult due to the relative 4°C hypothermic storage environment. Moreover, its long-established use offers logistical familiarity and ease of integration into standard procurement workflows.

Despite these advantages, SCS has significant limitations: temperature fluctuations and uneven cooling during transport can lead to inconsistent preservation, and the absence of pressure regulation may contribute to barotrauma and microvascular injury. One of the major disadvantages noted in the literature is that prolonged cold ischemia correlates strongly with the increased incidence of PGD and delayed graft function, especially when compounded by prior warm ischemia [16,17]. The compounded ischemia can exacerbate endothelial damage and impair alveolar function, reducing the likelihood of successful post-transplant recovery. Thus, although SCS remains a widely used and accessible method, its limitations have caused interest in more controlled and dynamic preservation approaches.

Selection criteria for DCD lung donors

Standard Criteria Donors

Given the heightened risk of graft injury, DCD donors have traditionally been subject to more stringent selection criteria than their DBD counterparts. Traditionally, donors under 55 years of age have typically been preferred for DCD lung procurement, though select programs extend criteria to include older donors based on functional evaluation via EVLP or OCS [12,13]. Per the EXPAND trial, most selection protocols require a PaO₂/FiO₂ ratio of >300 mmHg. However, in EVLP-supported programs, lungs with PaO₂ as low as 250 mmHg have been demonstrated to be safe for transplant. In addition, imaging plays an important role in selection: standard criteria donor (SCD) lungs should not demonstrate infiltrates or pneumonia on imaging and, if obtainable, bronchoscopy. Lungs demonstrating contusions may still be considered so long as the pO_2_ is within an acceptable range.

Extended Criteria Donors

Extended criteria donors (ECDs) are typically older individuals or those with an extensive smoking history (>20 pack-year), prolonged mechanical ventilation, or lower PaO_2_/FiO_2_ ratios. Nevertheless, the use of ECD-DCD lungs is becoming more common given the advances in perfusion technology. The EXPAND trial demonstrated that DCD lungs that were considered extended-criteria resulted in outcomes comparable to those from standard DBD donors when evaluated and preserved via machine perfusion. Similarly, institutional reports show successful use of ECD-DCD lungs in high-volume centers with rigorous assessment protocols [10].

Clinical outcomes: DCD versus DBD

**Figure 1:**
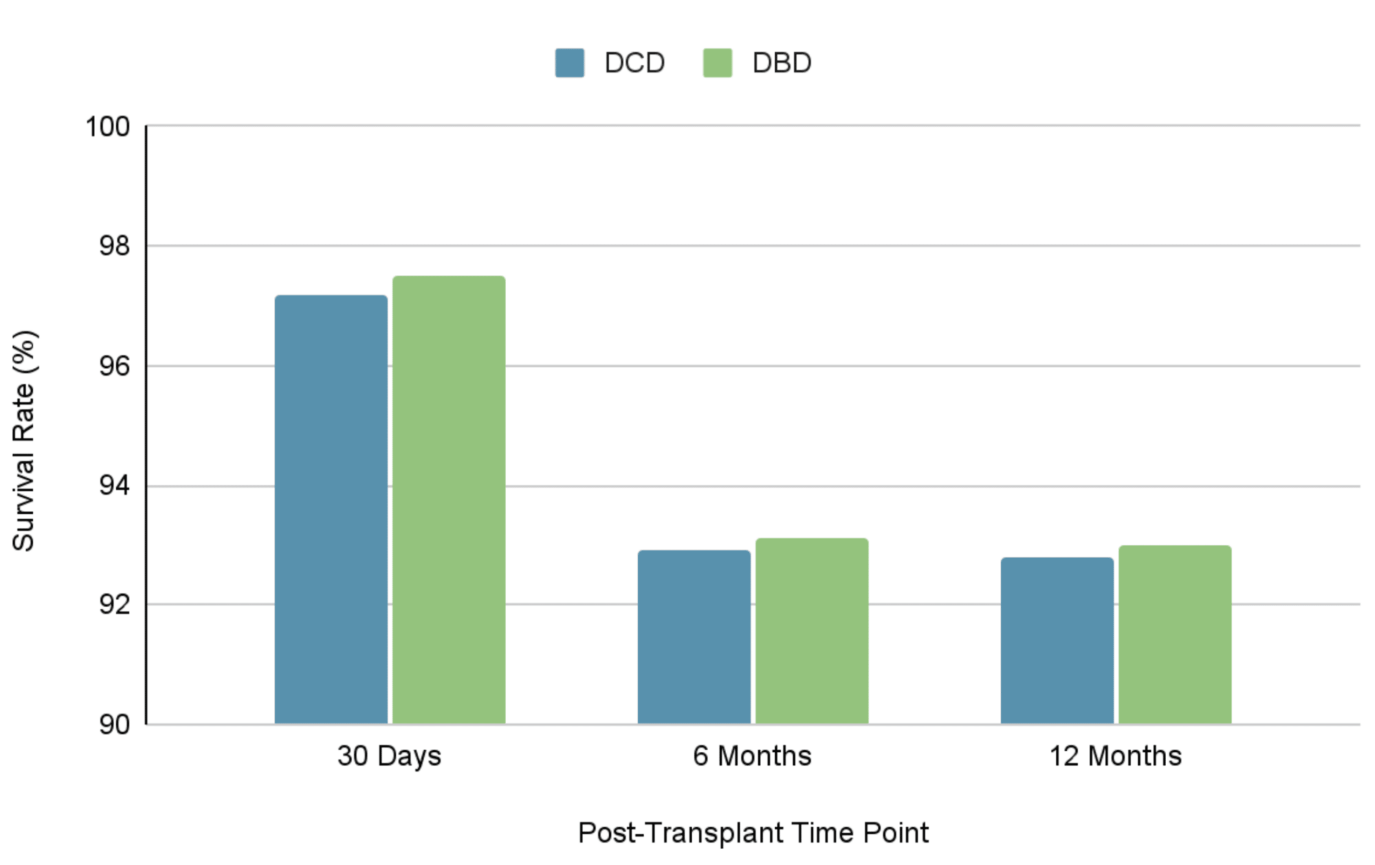
Survival rates of DCD and DBD lung transplant recipients over time DCD: donation after circulatory death; DBD: donation after brain death Survival rate data adapted from Spadaccio et al. [17]. Image credits: Author's own

Recent studies have provided an understanding of the clinical outcomes associated with DCD LTx in comparison to DBD. As shown in Figure 1, a meta-analysis conducted by Spadaccio et al. demonstrated a short-term (30 days to 12 months) survival advantage for DCD lung transplant recipients, despite a slightly higher initial mortality risk. Specifically, per Spadaccio et al., DCD recipients exhibited a 4.82% improved survival rate over five years compared to DBD recipients [17]. This finding suggests that while early postoperative management may be more complex in DCD cases, the long-term prognosis can be favorable.

As suggested by national registry data from the ISHLT, five-year survival rates, lack of chronic lung allograft dysfunction (CLAD), and bronchiolitis obliterans syndrome (BOS)-free survival were similar between DCD and DBD recipients [18].

Counterpoints and controversies

Operational and Logistical Challenges

While machine perfusion technologies such as OCS and XVIVO have revolutionized lung transplantation, their implementation presents real-world logistical barriers. The introduction of portable perfusion devices necessitates adjustments in procurement timing, transport logistics, and OR scheduling. Unlike static cold storage, which can be managed by a smaller surgical team, MP often requires equipment-specific trained perfusionists and remote monitoring support for when issues arise. Additionally, Wagner et al. highlighted that longer setup and perfusion times may delay implantation and increase the overall complexity of multi-organ recovery efforts [10].

Cost-Effectiveness and Resource Utilization

As mentioned in the previous section, current research highlights the clinical advantages of MP over SCS, though its implementation remains a significant financial investment. However, while machine perfusion systems such as the Organ Care System and XVIVO Perfusion System have significant costs, evidence indicates their cost-effectiveness through improved patient outcomes and expanded donor lung utilization. The upfront costs of these platforms are also typically offset by reductions in postoperative complications, shorter ICU stays, and increased transplant volumes.​ A study from the University Health Network in Ontario found that after implementing EVLP, transplants occurred more quickly without significantly increasing costs, suggesting that EVLP can improve efficiency without adding financial strains on high-volume centers with the infrastructure and staffing limitations, which makes such technology beneficial [19].​ Peel et al. found EVLP decreased the recipient waitlist time when compared to the period before incorporating EVLP into their practice [19].

Limited Standardization and Variability in Protocols

A major concern within the transplant community is the absence of universally accepted protocols for using EVLP and OCS systems. Institutions differ widely in their perfusion durations and assessment criteria (e.g., PaO₂/FiO₂ ratio thresholds) for transplant suitability, in turn complicating clinical trials, impeding cross-institutional data comparisons, and contributing to inconsistent patient outcomes [12]. However, the development of shared, evidence-based guidelines is in progress, with some efforts led by ISHLT. As MP becomes more commonplace, universal guidelines should be adopted.

Potential for Device-Related Complications

Despite its benefits, MP platforms such as the OCS and EVLP are not without risk. Wagner et al. describe technical issues, such as pump failure, tubing leaks, and perfusate contamination, that may compromise usability [10].

Concerns About Reconditioned Marginal Lungs

The ability of EVLP and OCS to rehabilitate marginal lungs has transformed donor selection, though many thoracic transplant clinicians remain hesitant. Krutsinger et al. noted that while early results are encouraging, long-term data on the performance of "reconditioned" lungs remains limited [10].

Future research

As the use of MP continues to evolve in DCD LTx, several key areas emerge as priorities for future investigation, one of the most prominent being normothermic regional perfusion (NRP). As NRP becomes more utilized, there is a clear need to explore the long-term outcomes of OCS following thoracoabdominal NRP in DCD lung transplants. Bashian et al. provided evidence suggesting comparable short-term survival and graft function between NRP and OCS groups, though superior three-year survival outcomes were seen in heart transplant recipients who underwent NRP compared to those who received only OCS-preserved organs [20]. These findings also highlight the importance of differentiating between the procurement technique (rapid recovery vs. TA-NRP) and the preservation platform (OCS vs. static cold storage) when designing future lung transplant studies. Additionally, the low utilization rate of lungs retrieved via TA-NRP, reported at only 11.6%-14.9%, underscores the need to investigate specific barriers to lung use following TA-NRP [21].

## Conclusions

While DCD LTx presents a promising opportunity to expand the lung donor pool, it also introduces significant challenges, primarily related to ischemic injury and organ viability. The conventional preservation method, SCS, has limitations in both the distance lungs can be transported and the duration for which they remain viable. Recent advancements in MP technologies, such as EVLP and OCS, have shown substantial promise in mitigating these issues by allowing for real-time assessment and reconditioning of marginal lungs, improving transplant success rates, and increasing organ utilization. Studies have demonstrated that DCD lung transplants when preserved with MP can yield comparable outcomes to DBD lungs, both in the short and long term. Moreover, the integration of MP technologies increases the prevention of ischemic injury, a key concern with DCD lungs. Despite these benefits though, the widespread adoption of these advanced technologies is limited by logistical, cost-related, and protocol standardization challenges. As transplant centers continue to gain experience and as protocols are refined, the potential for DCD lung transplantation to alleviate the chronic organ shortage becomes increasingly viable. Moving forward, expanding the use of MP in DCD lung transplantation could significantly increase the availability of suitable lung allografts, provided that logistical, financial, and operational challenges are addressed.
